# Clinical Significance of Amyloid Precursor Protein in Patients with Testicular Germ Cell Tumor

**DOI:** 10.1155/2013/348438

**Published:** 2013-04-14

**Authors:** Yuta Yamada, Tetsuya Fujimura, Satoru Takahashi, Kenichi Takayama, Tomohiko Urano, Taro Murata, Daisuke Obinata, Yasuyoshi Ouchi, Yukio Homma, Satoshi Inoue

**Affiliations:** ^1^Department of Urology, Graduate School of Medicine, The University of Tokyo, 7-3-1 Hongo, Bunkyo-ku, Tokyo 113-8655, Japan; ^2^Department of Urology, National Center of Global Health and Medicine, Shinjuku-ku, Tokyo, Japan; ^3^Department of Urology, Nihon University, School of Medicine, Itabashi-ku, Tokyo, Japan; ^4^Department of Geriatric Medicine, Graduate School of Medicine, The University of Tokyo, 7-3-1 Hongo, Bunkyo-ku, Tokyo 113-8655, Japan; ^5^Department of Anti-Aging Medicine, Graduate School of Medicine, The University of Tokyo, 7-3-1 Hongo, Bunkyo-ku, Tokyo 113-8655, Japan; ^6^Division of Gene Regulation and Signal Transduction, Research Center for Genomic Medicine, Hidaka-shi, Saitama, Japan

## Abstract

*Introduction*. The biological role of amyloid precursor protein (APP) is not well understood, especially in testicular germ cell tumors (TGCTs). Therefore, we aimed to investigate the immunoreactivity (IR) and expression of APP in TGCTs and evaluated its clinical relevance. *Materials and Methods*. We performed an analysis of immunohistochemistry and mRNA expression of APP in 64 testicular specimens and 21 snap-frozen samples obtained from 1985 to 2004. We then evaluated the association between APP expression and clinicopathological status in TGCTs. *Results*. Positive APP IR was observed in 9.8% (4/41) of seminomatous germ cell tumors (SGCTs) and 39.1% (9/23) of nonseminomatous germ cell tumors (NGCTs). NGCTs showed significantly more cases of positive IR (*P* = 0.00870) and a higher mRNA expression level compared with those of SGCTs (*P* = 0.0140). Positive APP IR was also significantly associated with **α**-fetoprotein (**α**FP) elevation (*P* = 0.00870) and venous invasion (*P* = 0.0414). *Conclusion*. We observed an elevated APP expression in TGCTs, especially in NGCTs. APP may be associated with a more aggressive cancer in TGCTs.

## 1. Introduction

Testicular cancer is a relatively rare cancer that accounts for approximately 1–1.5% of male cancers, and 90–95% of these cancers are testicular germ cell tumors (TGCTs) [1]. TGCTs can be classified into two major histological categories, namely, seminomatous germ cell tumor (SGCT) and nonseminomatous germ cell tumor (NGCT). NGCTs, which include yolk sac tumors, embryonal cell carcinomas, teratomas, and choriocarcinomas, are different from SGCTs with regard to clinical characteristics and therapy required.

Amyloid precursor protein (APP) is a type 1 transmembrane protein that is considered to play a key role in Alzheimer's disease. It has multiple isoforms attributable to alternative splicing and is expressed in various types of human cells. APP695 predominantly exists in the neurons whereas other isoforms such as the APP751 and APP770 are expressed in nonneuronal cells [2]. The biological role of APP is not well understood. APP and its cleaved forms have been suggested to mediate various functions, including cell adhesion [3], cell signaling [4], and cell growth [5–7]. These functions are important in carcinogenesis, and APP expression may be involved in the development of various cancers [8–13]. 

We have previously shown that APP is a primary androgen-responsive gene that promotes the growth of prostate cancer cells [14]. In the present study, we investigated APP immunoreactivity (IR) and APP mRNA expression in TGCTs and evaluated its clinical significance.

## 2. Materials and Methods

### 2.1. Patient Characteristics and Tissue Preparation

Sixty-four testicular specimens and 21 snap-frozen testicular samples were obtained from orchiectomies performed between 1985 and 2004. These samples were used for analysis of APP immunostaining and mRNA expression.

For APP immunostaining, 64 cancerous lesions and 31 benign testicular lesions were identified in 64 slides. The 64 cancerous lesions included 41 SGCTs and 23 NGCTs. NGCT lesions consisted of 3 embryonal carcinomas, 1 teratoma, 1 yolk sac tumor, and 18 mixed germ cell tumors (no case of pure choriocarcinoma). Of the 18 mixed TGCTs, 10 TGCTs consisted of both SGCT and other components of NGCT. Staging was performed according to the TNM 2009 staging system [1]. Patients with metastasis (12 cases) were also classified in terms of prognosis according to the International Germ Cell Consensus Classification (IGCCC) [15].

The age of the patients ranged from 0 to 71 years (mean, 35.2 years). Forty-two patients had pathological stage T1, and 22 patients had T2–T4. Mean levels of lactate dehydrogenase (LDH), beta human chorionic gonadotropin (*β*hCG), and *α*-fetoprotein (*α*FP) were 363 IU/mL, 614 ng/mL, and 431 ng/mL, respectively. Before surgery, no patients received chemotherapy or radiation. This study was approved by our institutional ethical committee. All the patients or their parents provided a written informed consent.

### 2.2. Immunohistochemistry

Immunohistochemistry for APP expression was performed by the streptavidin-biotin method as previously described [16]. Six-micrometer-thick sections were deparaffinized with 2 changes of xylene for 3 min each, then dehydrated using decreasing concentrations of ethanol, and rinsed in Tris-buffered saline (TBS). Antigen retrieval was carried out immersing the sections in citric acid buffer (2 mM citric acid and 9 mM trisodium citrate dehydrate, pH 6.0) and autoclaved at 121°C for 10 min. After a cooling period of 3 min, the sections were washed with TBS and blocked with endogenous peroxidase with 0.3% H_2_O_2_. The sections were then incubated in 10% bovine serum albumin (BSA) for 30 min. The slides were incubated overnight at 4°C with a primary rabbit polyclonal antibody for APP. For primary antibody, we applied 1 : 100 diluted rabbit polyclonal antibody for APP (number 2452, Cell Signaling Technology, Tokyo, Japan). After the sections were washed in TBS, they were incubated with Envision (DAKO, Carpinteria, CA, USA). The antigen-antibody complex was visualized with 3,3′-diaminobenzidine tetrachloride (DAB) solution (1 mM DAB, 50 mM Tris-HCl buffer, pH 7.6, and 0.006% H_2_O_2_). For negative controls, normal rabbit IgG was used instead of primary antibodies.

### 2.3. Antibodies

 Anti-APP antibody is a rabbit polyclonal antibody produced by immunizing rabbits with a synthetic peptide corresponding to residues surrounding the Thr668 of human APP695. Antibodies used in this study detect endogeneous levels of several isoforms of *β* amyloid precursor protein including APP695, APP770, and APP751 (number 2452, Cell Signaling Technology, Tokyo, Japan).

### 2.4. Immunohistochemical Assessment

Immunostained slides were evaluated for intensity scores as described in the previous literature [17]. The intensity score of immunostaining was rated from 0 to 3+ (0: none, 1: weak, 2: moderate, 3: strong), with 2+ or 3+ staining considered “positive immunoreactivity (IR)” for protein overexpression. A score of 1+ was considered the cut-off point because all normal testicular lesions showed an intensity score of 0 to 1+. Two observers (Y. Yamada and T. Fujimura) evaluated the slides, and a third observer (T. Murata) estimated the scores of the slides in case of disagreement between the 2 observers. 

### 2.5. RNA Extraction and Quantitative Reverse Transcription-Polymerase Chain Reaction

Total RNA was extracted from snap-frozen samples using ISOGEN reagent (Nippon Gene, Tokyo, Japan). First-strand cDNA was generated using PrimeScript (Takara, Kyoto, Japan). The resulting cDNA was subjected to real-time polymerase chain reaction (PCR) using an Applied Biosystems 7000 sequence detector system based on SYBR Green I fluorescence. mRNA expression was normalized for GAPDH mRNA levels. PCR protocol was as previously described [18]. Sequences of PCR primers are described below.

 GAPDH forward: GGTGGTCTCCTCTGACTTCAACA
 GAPDH reverse: GTGGTCGTTGAGGGCAATG
 APP forward: CACAGAGAGAACCACCAGCA
 APP reverse: ACATCCGCCGTAAAAGAATG.

### 2.6. Statistical Analyses

We used the statistical software JMP Pro version 9.0.2 (2010 SAS Institute Inc.) for data analysis. The chi-square test (*χ* ^2^) and Fisher test were used for analysis of association between APP IR and clinicopathological characteristics such as tumor stage; lymph node stage; clinical stage; pretreatment serum levels of LDH, *α*FP, and *β*hCG; and prognostic classification (IGCCC 1997). Students' *t-*test and Welch test were used for the evaluation of APP level in compared histological groups. Mann-Whitney *U* test was used to analyze statistical differences in the relative levels of APP mRNA between SGCTs and NGCTs. Log-rank test was performed to analyze the statistical difference of cancer-specific survival between patients with a SGCT and those with an NGCT. A *P* value of <0.05 was considered statistically significant.

## 3. Results

### 3.1. APP Immunostaining in Normal Testicular Tissue and Testicular Germ Cell Tumor

**Figure 1:**
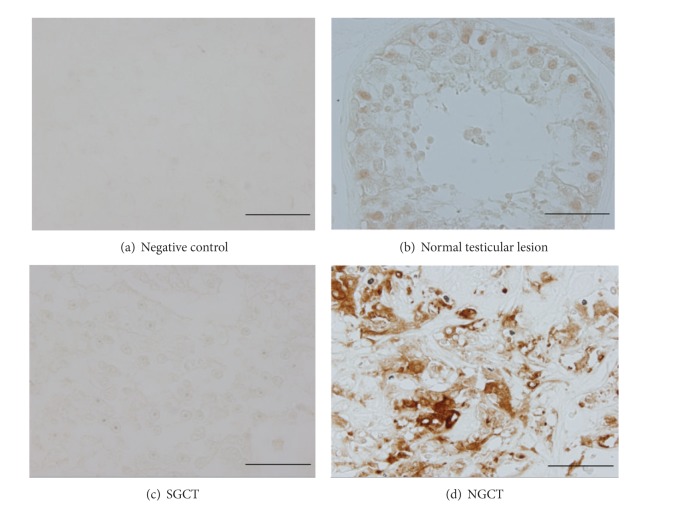
Immunohistochemistry of amyloid precursor protein (APP) in testicular tissue specimens. (a) Negative control showing negative immunostaining. (b) Normal testicular lesion showing weak staining (intensity score: 1). (c) Seminomatous germ cell tumor lesion showing negative immunostaining (intensity score: 0). (d) Nonseminomatous germ cell tumor lesion (embryonal cancer component) showing strong APP immunostaining (intensity score: 3). *Scale bars*, 50 *μ*m.

Majority of the patients with benign testicular lesion (80.6%) and SGCT (87.8%) had no immunostaining for APP (Table 1 and Figure 1(c)). Six of 31 a benign testicular lesions (Figure 1(b)) and 1 of 41 SGCTs showed weak immunostaining, with an intensity score of 1+. Four of 41 SGCTs (9.8%) and 9 of 23 NGCTs (39.1%) (Table 1) showed an intensity score of 2+ to 3+ (Figure 1(d)), and these lesions were considered as showing “positive immunoreactivity (IR).” Positive IR was not seen in SGCT components of the 10 mixed TGCTs.

The rate of positive APP IR was significantly higher in NGCT lesions compared with those in SGCT and in benign testicular lesions (*P* = 0.0001). When compared by pairs, positive APP IR was more frequently observed in NGCT lesions than in SGCT (*P* = 0.00870), although no significant differences were found between SGCT and benign testicular tissues (Table 2). Positive APP IR was not associated with tumor stage or lymph node stage. However, it was associated with elevated *α*FP level (*P* = 0.00870) and venous invasion (*P* = 0.0414). Other tumor markers such as LDH and *β*hCG did not show association with positive APP IR. 

In all, cancer-specific death occurred in 3.1% (2/64) of the whole population. There was no significant difference in cancer-specific survival (*P* = 0.495; data not shown) and IGCCC (*P* = 0.222, Table 2) between SGCTs and NGCTs. 

### 3.2. APP mRNA Expression in Testicular Germ Cell Tumors

**Figure 2:**
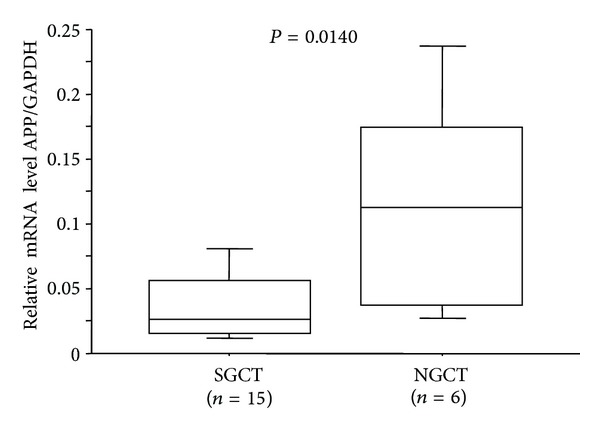
Quantitative RT-PCR analysis of amyloid precursor protein (APP) in seminomatous germ cell tumors (SGCTs) and nonseminomatous germ cell tumors (NGCTs). The figures in box and whisker plot show the relative mRNA levels of APP quantified by normalization to GAPDH mRNA levels. *Bars*, ±SDs. A statistically significant difference in APP expression was observed between SGCTs and NGCTs (*P* = 0.0140).

Quantitative RT-PCR analysis was performed to evaluate the relative APP mRNA levels in SGCT (*n* = 15) and NGCT (*n* = 6) tissues. We observed significantly higher levels in NGCT than in SGCT (*P* = 0.0140, Figure 2). 

## 4. Discussion

APP is ubiquitously expressed in various types of human cells. The extracellular domain of the APP has been suggested to be involved in transcellular adhesion [3] and neurite outgrowth [19, 20]. It may also function as a cell surface receptor [21, 22]. The intracellular domain of the APP is considered to be involved in cell migration [21, 23], cell signaling [4, 21], and apoptosis [21, 24, 25]. It also activates proliferation of epithelial cells [6]. These lines of evidence suggest that APP is involved in cancer cell proliferation. We have previously reported that APP contributes to androgen-dependent proliferation of prostate cancer cells and that the rate of cancer-specific survival for patients with APP-positive tumors was lower than that for patients with APP-negative tumors [14]. Another study revealed an APP overexpression in human pancreatic and colon cancer [13]. In the same study, small interfering RNA-mediated knockdown of APP also resulted in decreased cancer cell growth [13]. Involvement of APP expression is also suggested in cancer cells originating from the nasopharynx [8], oral cavity [9], thyroid [10, 11], and colon [12]. In a testicular germ cell tumor (TGCT), a recent study suggested an association of APP expression in transformed human pluripotent stem cells [26]. However, clinical significance of APP expression has not been evaluated. Therefore, we investigated the expression of APP in TGCTs and evaluated its association with clinical characteristics of TGCT.

The present study shows that APP is more strongly expressed in NGCTs than in SGCTs. In terms of APP IR, we found a greater rate of positive cases in NGCTs than in SGCTs (39.1% versus 9.8%). APP mRNA levels were higher in NGCTs than in SGCTs. In a study by Venkataramani et al. [26], APP immunostaining was positive in both undifferentiated SGCTs and embryonal cell carcinomas. However, when mRNA levels were compared among cell lines such as TCam-2 (SGCT cell line), NCCIT (mixed cell line of SGCT and NGCT), and NTera-2 (embryonal carcinoma cell line), significantly higher expression levels were observed in the NTera-2 cell line.

Venous invasion, a risk factor for occult metastases of TGCT [27], was also significantly associated with positive APP IR. Together with the result of immunostaining and mRNA analysis, our findings show that APP expression is associated with NGCTs and venous invasion, and that APP may be related to a more aggressive carcinogenesis, thus indicating that APP may be a potential prognostic marker of TGCTs. 

 In the present study, no significant differences were observed in cancer-specific survival between APP-positive and APP-negative cases in a TGCT. Because TGCTs are highly responsive to chemotherapy or radiation therapy, cancer-specific death occurred in only 3.1% (2/64) of the whole study population. In addition, no patient was classified in the poor-risk group in IGCCC in this study. For this reason, we may not have observed significant differences in survival. However, a trend toward an association between APP IR and a higher-risk group in IGCCC were observed. In a study involving a larger patient population, positive APP IR might show a significant association with a worse-survival or a higher-risk group in IGCCC.

The mechanism of APP expression in cancer cells is not fully understood. Growth factors such as EGF and PDGF promote APP cleavage in a Ras-dependent pathway [28]. In nasopharyngeal cancers, APP expression is regulated by epithelial growth factor receptor (EGFR) activation [8]. Because EGFR activation is considered to play an important role in the pathogenesis of many human malignancies [29], APP may be linked to carcinogenesis. Several lines of evidence revealed overexpression and activation of EGFR in NGCT. A study evaluating 24 TGCT cases for EGFR expression showed a strong association between *β*hCG-expressing component of NGCT and EGFR 1 with HER-2/neu coexpression [30]. In this study, expression of the TGF-*α*, a known EGFR ligand, was also found in 36% of EGFR-positive NGCT cases. Another study showed Her-2/neu overexpression in 24% of the NGCT cases [31]. EGFR activation in NGCTs and a potential role of EGFR-regulated APP expression in some of the malignancies support the possibility of EGFR-mediated APP expression in NGCTs. 

## 5. Conclusions

The current findings show that APP levels are elevated in TGCTs, especially in NGCTs. APP elevation may indicate a more aggressive histological type of TGCTs.

## Figures and Tables

**Table 1:** Immunoreactivity of APP in human testicular specimens.

APP intensity score	Number of cases (%)		
	Normal testicular tissue	SGCT	NGCT
**0**	**25 (80.6) **	**36 (87.8) **	**14 (60.9) **
**1+ **	**6 (19.4) **	**1 (0.02) **	**0 (0) **
2+	0 (0)	3 (0.07)	5 (21.7)
3+	0 (0)	1 (0.02)	4 (17.4)

Total	31	41	23

APP: amyloid precursor protein, SGCT: seminomatous germ cell tumor, NGCT: nonseminomatous germ cell tumor. Intensity score was rated from 0 to 3+ and defined as 0: no immunostaining, 1+: weak intensity, 2+: moderate intensity, 3+: strong intensity. “0” and “1+” are defined as “negative immunoreactivity.” “2+” and “3+” are defined as “positive immunoreactivity.” Numbers in boldface indicate negative immunoreactivity while those in light face indicate positive immunoreactivity.

**Table 2:** Relationships between APP immunoreactivity and clinicopathological characteristics in TGCT patients (*n* = 64).

Clinicopathological data	APP immunoreactivity		
	Negative (*n* = 51)	Positive (*n* = 13)	*P* value
Age (years ± SD)	36.5 ± 10.7	30.0 ± 15.8	0.182
Tumor marker			
LDH			0.340
High	20	7	
Normal	31	6	
*α*FP			0.00870
High	14	9	
Normal	37	4	
*β*hCG			0.844
High	29	7	
Normal	22	6	
IGCCC			0.222
Good	6	1	
Intermediate	2	3	
Poor	0	0	
Stage			
T stage			0.0978
T1	36	6	
T2–4	15	7	
N stage			0.444
N0	43	9	
N1–3	8	3	
Unknown	0	1	
Venous invasion			0.0414
Positive	7	5	
Negative	44	8	
Pathology			0.0001
Benign testicular lesion	31	0	
SGCT	37	4	
NGCT	14	9	
Compared histological types			
SGCT versus NGCT	—	—	0.00870
SGCT versus benign testicular lesion	—	—	0.129
NGCT versus benign testicular lesion	—	—	0.0002

APP: amyloid precursor protein, TGCT: testicular germ cell tumor, LDH: lactate dehydrogenase, *α*FP: alpha fetoprotein, *β*hCG: human chorionic gonadotropin *β* subunit, IGCCC: International Germ Cell Consensus Classification, SGCT: seminomatous germ cell tumor, NGCT: nonseminomatous germ cell tumor.

## Abbreviations

APP: Amyloid precursor protein
TGCT: Testicular germ cell tumor
qRT-PCR: Quantitative reverse transcription polymerase chain reaction
SGCT: Seminomatous germ cell tumor
NGCT: Nonseminomatous germ cell tumor
IR: Immunoreactivity
*α*FP: Alpha fetoprotein
IGCCC: International Germ Cell Consensus Classification
LDH: Lactate dehydrogenase
*β*hCG: Beta human chorionic gonadotropin
EGFR: Epithelial growth factor receptor.
